# From myth to machine: artificial intelligence and the future of organ transplantation

**DOI:** 10.3399/BJGPO.2025.0158

**Published:** 2026-02-11

**Authors:** Arun Dahil, Cameron Kumar Pinn

**Affiliations:** 1 Primary Care Research Centre, University of Southampton, Southampton, UK.

**Keywords:** Surgery, Artificial Intelligence, Transplant Medicine, General Practitioners

## Introduction

In ancient Roman mythology, the tale of Mercury and the lyre tells of the trickster god transforming a tortoise shell and the stolen entrails of one of Apollo’s cattle into the first lyre, the music from which ultimately appeases Apollo, the god of music and medicine. In doing so, Mercury turned the remnants of death into an instrument of harmony. This imagery resonates with organ transplantation: the end of one life can become the beginning of another.

Transplantation represents one of modern medicine’s most extraordinary achievements, yet its success remains constrained by persistent challenges: organ scarcity, uncertainty of graft viability, and the fragility of clinical decision-making. Today, artificial intelligence (AI) may offer the transformative capacity to address these challenges. AI could help turn loss into life, and hesitation into hope.

This editorial considers the role of AI in the future of transplantation medicine, with particular attention to its implications for organ assessment, allocation, and post-operative care. While many of these developments emerge from secondary and tertiary settings**,** their downstream effects will be deeply felt in primary care. As the first point of contact for chronic disease management, immunosuppressant titration, and early detection of complications, general practice must evolve in tandem with these innovations.

### AI in organ preservation and assessment

Significant progress has been made in preserving organs outside the body, most notably through ex vivo normothermic and hypothermic perfusion technologies. These systems can maintain donor organs for extended periods while enabling real-time physiological monitoring of perfusate parameters such as lactate clearance, transaminase levels, and pH, all correlated with graft viability.^1^

These streams of data, while valuable, are complex to interpret manually. For this task, AI possesses a unique advantage. Machine learning models trained on perfusion data can integrate dozens of variables to generate real-time viability assessments. In one study, the InsighTx model demonstrated 85% accuracy in predicting successful lung transplantation and 90% accuracy in flagging unsuitable organs, outperforming specialist review alone.^2^ Retrospective validation revealed that the model could significantly reduce the use of non-viable lungs and increase the utilisation of marginal organs.

AI-based tools are also under development for liver and kidney transplantation.^3,4^ These technologies may soon assist transplant teams in determining organ suitability with objective, data-driven insight. For GPs managing those who have received transplant in the community, understanding how grafts were selected and preserved, especially when marginal organs are used, can provide valuable clinical context. For example, if a GP knows that a transplanted organ had borderline perfusion metrics, early signs of graft dysfunction may be investigated more urgently. Such context may be crucial when patients present with unexplained graft dysfunction or delayed recovery.

### Smarter allocation: towards dynamic organ sharing networks

Beyond organ viability, AI also promises to reshape the broader logistics of organ allocation. Current allocation algorithms rely on relatively static criteria: blood type, human leukocyte antigen match, recipient urgency, and geography. These rules, while essential, lack flexibility and often fail to accommodate the complexity and nuance of modern transplantation medicine. AI-assisted allocation systems such as Smart Match are being developed to address these shortcomings, using vast arrays of patient and organ data to predict optimal pairings and long-term outcomes.^5^ These models could facilitate a more dynamic allocation process, potentially spanning national or even European borders. A kidney retrieved in Manchester could, theoretically, be matched to a recipient in Milan within seconds, with AI accounting for logistics, recipient health status, immunological compatibility, and even transport infrastructure.

However, the promise of AI allocation must be balanced with caution. Rigorous validation is needed to ensure equity, transparency, and resistance to algorithmic bias. These issues are not abstract. GPs, who build long-term relationships with patients, are often the first to encounter the ethical tensions arising from opaque decisions or perceived unfairness. Engaging GPs in discussions around data justice and patient communication will be vital to sustaining trust in an AI-integrated transplant ecosystem.

### AI in the operating room and beyond

Within the surgical environment, AI is already augmenting human ability. Real-time decision support tools, robotic assistance, and augmented reality (AR) overlays are under investigation for their potential to guide surgeons with anatomical precision and physiological feedback.^5^ These technologies may one day provide intraoperative guidance akin to autopilot systems in aviation; helping surgeons navigate complex anatomy or alerting them to signs of graft compromise. For example, intraoperative AI could generate risk scores or notes that accompany the surgical report. If an AI system flags a higher-than-average risk of complications during surgery, that information could be communicated to the GP in the discharge summary, influencing how post-operative care is managed.

Beyond the operating room, AI’s most profound impact may lie in transforming post-operative care. Wearable sensors can feed real-time data into predictive models, detecting early signs of rejection, infection, or adverse drug reactions. Tailored immunosuppressive regimens may be developed based on personalised biomarker analysis, integrated into AI-powered decision systems.

This transition from episodic to continuous monitoring would place new demands on primary care. GPs may be tasked with interpreting alerts, adjusting medications, and coordinating with tertiary teams. For example, if an AI-driven alert detects a pattern suggestive of early rejection (such as rising serum creatinine), the GP may promptly order additional tests, adjust immunosuppressive medications like tacrolimus, or consult the transplant team for further advice. The continuous data stream will require GPs to integrate AI-generated recommendations into routine patient management. Investigators are already training deep learning systems to interpret biopsy data and generate early warnings of rejection.^6^ Soon, such systems may interface directly with GP electronic health records, prompting timely reviews or investigations. As these tools move into practice, GPs must not be passive recipients of AI outputs; they must be empowered to critically appraise and act on these insights.

However, realising this vision will require overcoming practical barriers. Many GP practices may lack the necessary digital infrastructure to integrate continuous monitoring systems, and clinicians often have limited training in interpreting complex AI outputs. Moreover, constant streams of data can generate frequent alerts, risking 'alert fatigue' among busy doctors. Addressing these hurdles, through investment in technology, training, and smarter alert management, will be essential to ensure that AI truly augments, rather than burdens, primary care.

### The patient at the centre

The technological capacity of AI is immense, but its adoption must be guided by ethical clarity. Patients must be assured that their data, the lifeblood of AI systems, are used responsibly. Transparency, informed consent, and robust data protection are vital to earning and maintaining public trust.

GPs have a central role as mediators and interpreters. With their unique access to patients’ longitudinal stories and psychosocial contexts, GPs are well-placed to bridge the gap between patients and emerging technologies. As AI becomes more embedded in care decisions, GPs can support patients in understanding how these systems influence their treatment and can advocate for human-centred oversight where clinical nuance risks being lost in algorithmic abstraction.

Above all, AI must not depersonalise care. Transplantation is not merely a technical feat; it is a deeply human act, interwoven with hope, grief, and gratitude. AI must therefore support, not supplant, clinical judgement and patient-centred dialogue, a principle that resonates especially within the generalist ethos of primary care.

### A call to action

Transplantation offers one of the most profound gifts in medicine: life from death. However, this gift is often jeopardised by scarcity, uncertainty, and inefficiency. AI holds the potential to address all three.

When technology exists, the clinical need is urgent, the ethical imperative is clear: it is time for primary care and surgical communities alike to engage actively with AI. Not as a replacement, but rather a partner in care.

To help GPs, specific actions could include:

- NHS policy reforms to provide GPs with protected time to augment their digital literacy learning through online and in-person courses, to understand and evaluate AI-driven insights.
- Primary care research organisations could arrange focus group discussions involving transplant patients and GPs about how AI could influence patient treatment.
- Publication of transparent guidelines, governance, and ethical outlines of the role of AI integrated systems within primary care.

By taking these steps, GPs can help ensure that AI enhances patient care without sacrificing the human touch at the core of medicine.

## Data

N/A
